# Dynamic sporulation gene co-expression networks for *Bacillus subtilis* 168 and the food-borne isolate *Bacillus amyloliquefaciens*: a transcriptomic model

**DOI:** 10.1099/mgen.0.000157

**Published:** 2018-02-09

**Authors:** Jimmy Omony, Anne de Jong, Antonina O. Krawczyk, Robyn T. Eijlander, Oscar P. Kuipers

**Affiliations:** ^1^​ Laboratory of Molecular Genetics, University of Groningen, 9747 AG Groningen, The Netherlands; ^2^​ Top Institute Food and Nutrition (TIFN), Nieuwe Kanaal 9A, 6709 PA Wageningen, The Netherlands; ^3^​ NIZO Food Research, B.V., P.O. Box 20, Ede 6710 BA, Ede, The Netherlands

**Keywords:** sporulation, stages of sporulation, gene co-expression network, RNA-Seq, *Bacillus subtilis*, *Bacillus amyloliquefaciens*

## Abstract

Sporulation is a survival strategy, adapted by bacterial cells in response to harsh environmental adversities. The adaptation potential differs between strains and the variations may arise from differences in gene regulation. Gene networks are a valuable way of studying such regulation processes and establishing associations between genes. We reconstructed and compared sporulation gene co-expression networks (GCNs) of the model laboratory strain *Bacillus subtilis* 168 and the food-borne industrial isolate *Bacillus amyloliquefaciens*. Transcriptome data obtained from samples of six stages during the sporulation process were used for network inference. Subsequently, a gene set enrichment analysis was performed to compare the reconstructed GCNs of *B. subtilis* 168 and *B. amyloliquefaciens* with respect to biological functions, which showed the enriched modules with coherent functional groups associated with sporulation. On basis of the GCNs and time-evolution of differentially expressed genes, we could identify novel candidate genes strongly associated with sporulation in *B. subtilis* 168 and *B. amyloliquefaciens*. The GCNs offer a framework for exploring transcription factors, their targets, and co-expressed genes during sporulation. Furthermore, the methodology described here can conveniently be applied to other species or biological processes.

## Data Summary

We provide three supplementary figures and eight supplementary tables. Data used for the network reconstruction (with corresponding citations to their sources) and additional output files from the analysis are summarised in five extra files. This material and corresponding links to the files are available for download in the online version of this article (Supplementary Material).

**Impact Statement**

As a survival strategy, bacterial cells can adapt quickly to respond to harsh environmental conditions. Knowledge of how the genes controlling the sporulation stages are regulated is important for understanding the dynamics of sporulation behavior of bacteria, not only in model laboratory strains but also in industrial or environmental isolates. Our work explores gene co-expression networks (GCNs) that enable us to search for genes with similar expression profiles and gene ontologies (e.g., biological processes). This is crucial for transcending knowledge of the sporulation behavior of the model laboratory strain *B. subtilis* 168 and for expanding it to industrially significant strains like the food-borne isolate *B. amyloliquefaciens*. The networks provide a valuable data mining platform for genes of interest, in particular, those associated with sporulation.

## Introduction

Regulation of sporulation in Gram-positive bacteria is a tightly controlled process that can be divided into various stages. In *Bacillus subtilis,* sporulation involves regulatory elements such as the master transcriptional regulator of sporulation initiation Spo0A, sigma factors (σ^A^, σ^H^, σ^F^, σ^E^, σ^G^ and σ^K^) and several auxiliary transcriptional regulators active at the different stages of sporulation [1, 2]. Sporulation gene expression is well-documented for *B. subtilis* [3, 4] and for some Clostridia like (*Pepto*)*Clostridium difficile* [5, 6]. Sporulation morphogenesis leads to the formation of resistant forms of life, i.e., bacterial spores, which are encased by two protective layers, the peptidoglycan cortex and the proteinaceous coat [7]. After asymmetric cell division, two cellular compartments emerge, a larger mother cell and a smaller forespore, which will then follow different interdependent developmental pathways. During sporulation a cascade of compartment-specific RNA polymerase sigma (σ) factors regulate gene expression. The production and activation of these sigma factors are tightly controlled [8, 9].

Mature spores are protected against environmental stressors such as radiation, heat, oxidation and desiccation. Under favorable conditions, germination is initiated and followed by a series of events which leads to restoration of vegetative cell growth [3]. Some efforts have been made to investigate the sporulation-related regulatory cascades and their influence on phenotypic traits of the resultant spores [10–12]. However, much remains unknown about the genomic-scale organization of the sporulation regulatory network, especially for environmental isolates of members of the genus *Bacillus*. To explore this, we used undirected weighted gene co-expression networks (GCNs) to investigate the sporulation regulatory network of *B. subtilis* 168 and that of the food-borne strain *Bacillus amyloliquefaciens* B4140. A GCN is a graphical structure with genes (depicted as nodes) and edges (as the association between genes). Fundamentally, a node corresponds to a gene expression profile and an edge exists between two nodes in a network if the corresponding genes have similar expression profiles. GCNs provide essential data mining platforms for exploring the association between genes and their transcription factors. Such associations intrinsically represent the influence of metabolites and proteins. It is essential that the associations encoded in the edges are not confused with bi-directional regulatory arrows between adjacent nodes in a network since co-expression networks are undirected graphs.

Unlike gene regulatory networks, which connect transcription factors and non-coding RNAs to their targets, GCNs do not indicate regulatory effects, but rather show genes with similar expression profiles grouped in the same network vicinity. Fundamentally, GCNs do not distinguish between direct and indirect regulatory interactions and they miss gene neighborhood maps in the conventional cluster analysis [13]. Various modeling efforts have been made to understand molecular mechanisms and to predict sporulation behavior in *B. subtilis* [1, 12] and more recently to describe the transcriptional regulatory network of *B. subtilis* [14, 15]. We do not use kinetic modeling here, which was used to study mechanistic details of sporulation sub-networks, such as, the phosphorelay [11], master regulator Spo0A [16] and sporulation initiation [17]. We used RNA-Seq data from *B. subtilis* 168 and *B. amyloliquefaciens* to reconstruct, infer and compare their sporulation GCNs and underlying variations in gene regulatory mechanisms. Key regulatory factors are sometimes determined and validated experimentally, e.g., in the work of Arrieta-Ortiz *et al*. [14]; however, computational methods also aid the determination of candidate transcription factors and their target genes. To investigate the sporulation regulatory network, we explored gene clusters within the strains, assessed differences in GCNs between the two strains, and evaluated time-dependent progression of sporulation. The detected modules, which are defined as clusters of highly interconnected genes were manually curated and subjected to a gene set enrichment analysis [18]. This analysis revealed coherent functional gene classes within the network modules enriched in various processes. Here, we focus particularly on genes and modules associated with sporulation. The reconstructed GCNs were bench-marked with gene clusters obtained from *k*-means clustering analyses of the transcriptome analysis webserver for RNA-Seq expression data (T-REx) [19] which showed a good correlation between clusters and network modules.

We also study Spo0A, which is an activator of *sigE* expression. Under most conditions, higher expression levels of *sigE* are associated with higher expression levels of *spo0A*. However, it should first be noted that Spo0A is activated by phosphorylation so transcription of *spo0A* does not directly indicate the level of active phosphorylated Spo0A (Spo0A~P) in the cell. Spo0A represses *spo0A* expression [20] in addition to being regulated by other transcription factors (and/or sigma factors). The list of candidate genes from our networks can then be used for further research on regulatory mechanisms of interest (e.g., by knock-out studies, up/down-regulation) for specific transcription factors and their targets to assess the effect on gene expression levels. Moreover, a comparison of the sporulation transcription behavior of the listed genes in individual strains could provide clues on the potential differences in the strains’ sporulation pathways. A major setback in reconstructing high-quality GCNs is that they miss connections between the transcriptional factors and their target genes in the systems in which binding of the transcription factors does not result in gene transcription changes in the assessed conditions [21], while other processes regulate gene expression independent of transcription [22]. Such mechanisms that cannot be depicted by GCNs commonly occur in the genome-scale regulation of transcription networks.

## Methods

### Primary data for the network reconstruction

Publicly available *B. subtilis* 168 tiling array data from Nicolas *et al*. [23] was used as the primary transcriptome data (File S1, available in the online version of this article). All the genes and conditions in the dataset were used as input in the network reconstruction in the Sparse PArtial Correlation Estimation (SPACE) [24]. SPACE is a robust method for generating GCNs and it has been shown elsewhere to yield enriched modular networks [25, 26]. In SPACE, determination of the GCN structure is based on partial correlations in the network adjacency matrix.

### Experiments and secondary data for the network reconstruction and strains

The transcriptional (RNA-Seq) data for *B. subtilis* 168 and *B. amyloliquefaciens* analyzed in our work was obtained as described by Krawczyk [27] (deposited at NCBI, GSE108659). Briefly, sporulation of the strains was induced by the resuspension method [23], in which transfer of the bacterial cultures from a medium rich in nutrients to a poor medium initiates the sporulation response. The *Bacillus* cultures were grown at 37 °C with shaking (200 r.p.m.) in the casein hydrolysate (CH) medium till they reached an OD_600_ of 0.6. Subsequently, cells were collected by centrifugation, the CH medium was removed and the cultures were resuspended in the same volume of the pre-warmed Sterlini–Mandelstam (SM) medium. Culture samples of 300 µl and 15 ml were collected (at various time-points, reflecting the different stages of sporulation) by centrifugation for microscopic analysis and RNA isolation, respectively. Samples for each strain were obtained from two independent sporulation experiments.

For microscopic analysis, the collected cells were washed in PBS, fixed with preservation of cell membranes by use of 4 % paraformaldehyde and stored at −20 °C [28]. The progress of sporulation was examined for the fixed cells collected at different time-points, which were placed on a 1.0 % agarose microscopy slide supplemented with the 2 µg ml^−1^ membrane dye FM1-43 (Invitrogen), using phase-contrast microscopy [27]. The results of the microscopic analyses were used for selection of samples for RNA isolation that reflect various phases of sporulation (classified as P1 to P6, where P1 indicates cells before asymmetric division; P2 asymmetric cell division; P3 ongoing engulfment of the forespores by the mother cell compartments; P4 sporulating cells with phase-dark forespores; P5 ongoing phase transition of forespores from phase-dark to phase-bright; P6 sporulating cells with phase-bright forespores and released mature spores). As the two analysed strains did not sporulate at the same rates, they reached the respective sporulation phases at different time-points, i.e., 1, 2, 3, 4, 5 and 7 h in the case of *B. subtilis* 168 and 1.5, 3, 4, 5, 6, 7 and 8 h for *B. amyloliquefaciens* [27]. Additionally, the P0 samples that corresponded to the time-point immediately after the transfer of the bacterial cultures from the CH medium to the SM medium were included in the transcriptomic analysis. Total RNA was isolated by phenol:chloroform extraction and precipitation with ethanol and sodium acetate [29]. The RNA samples were subjected to next-generation directional sequencing on an Ion Proton Sequencer at the PrimBio Research Institute (Exton, PA, USA).

The RNA sequence reads were processed as described previously [27]. Briefly, the RNA sequencing reads were mapped to the reference genome of *B. subtilis* 168 using Bowtie2 [30]. The gene (RNA) expression values were generated as Reads Per Kilobase per Million reads (RPKM). The average RPKM values, which were calculated based on the results of two independent sporulation experiments, were used in the analysis.

The differentially expressed genes (DEGs) were determined per strain [19] and the data from the subsequent stages (P1 to P6) were normalized using P1 as a reference (contrast of five time-points). Time-point P0 corresponds to the state prior to the onset of sporulation. Progression of sporulation was inferred from quantitative analysis of the expression of major transcription factors that initiate and/or control sporulation. The analysis offers clues on variation in sporulation transcriptional behavior between the strains and differences in gene expression for groups of genes, especially those linked to sporulation. To benchmark the reconstructed networks, a list of genes in regulons of *B. subtilis* was downloaded from SubtiWiki (http://subtiwiki.uni-goettingen.de/) [31] and SporeWeb [3] and then mapped onto the GCN. File S2 and File S3 contain the data for the secondary network reconstruction corresponding to *B. subtilis* 168 and *B. amyloliquefaciens*.

### Reconstruction of *B. subtilis* 168 sporulation network

Genes showing similar expression profiles are often considered more likely to be connected, regulated by the same transcription factors and involved in the same biological function [32]. The assignment of edges (or connections) between genes in the network is based on guilt-by-association, as determined by using Pearson’s or Spearman rank correlation [33, 34]. Robustness of distance measures is based on the similarity matrices from gene expression data [35]. Pruning was used to remove weak non-significant entries in the adjacency matrix [36]. This process is the equivalent of deleting weak edges between nodes in a network. The resultant adjacency matrix represents the network structure on which module detection, gene set enrichment analysis and any down-stream processes are performed [37].

GCNs are commonly generated using methods based on partial correlations [24, 38] and weighted correlations [39, 40]. However, the structure and biological enrichment of a network are broadly influenced by transcriptome data quality and size. Therefore, it is essential to use high-quality data of sufficient quantity to generate biologically informative networks. Additional to using robust reconstruction methods, having less noisy data and a large number of experimental conditions is essential for improving co-expression analysis and network inference by reducing the likelihood of assigning false edges between nodes in a network. de Hoon *et al*. [1] investigated the hierarchical evolution of sporulation networks in bacteria and concluded that grasping the logic in the evolution of a model organism enables a better understanding of networks in closely related species, particularly at the functional organization level.

The most commonly used measures of association to assign edges between two genes (or nodes) in a network are the Pearson’s and Spearman’s rank correlation coefficient.

### Validating the *B. subtilis* 168 sporulation network

To validate the sporulation network, a list of DEGs from *B. subtilis* 168 obtained from RNA-Seq data obtained previously [27] was projected on the GCN and their spatial distribution on the network was assessed. The statistical language R version 3.2.2 (igraph library) was used to analyze the network modularity (*Q*) [41, 42]. Modularity is defined as the number of edges falling within groups minus the expected number in an equivalent network with random edge placements [43]. Generally, GCNs with high modularity (*Q*≈1) provide an optimal arrangement of edges in a network for which genes with similar expression profiles are grouped in modules. Essentially, GCNs with *Q*≈1 have dense connections between the nodes within specific modules, but they characteristically have sparse connections between the nodes in different modules.

### Mapping sporulation genes on the sporulation networks

We used SporeWeb [3] as a resource and reference for sporulation-associated genes and gene classes. The classes include genes involved in germination, genes encoding SpoVA proteins, cortex hydrolysis, sporulation-specific (SASP), cortex, coat, coat maturation, main regulation, transcription and phosphorelay. The genes in these classes were mapped onto the reconstructed GCNs to enable identification of other genes highly correlated to the sporulation process.

### Detection and significance of network modules and hubs

Genes that constitute hubs generally exhibit characteristic expression profiles representative of those in the modules to which they belong [13]. Sporulation regulatory networks in *B. subtilis* 168 have a modular architecture [1]. We assessed how the genes are connected to each other in the co-expression network using the R package walk–trap module detection method [44, 45]. Subsequently, the detected modules in the *B. subtilis* 168 GCN were manually curated: genes from two completely detached modules from large highly connected parts of the network or any other modules were merged into single modules.

### Identifying sporulation gene clusters from RNA-Seq data of *B. subtilis* 168 and *B. amyloliquefaciens*

To categorize genes into functionally related groups, gene clusters were determined using *k*-means clustering [46]. The number of gene clusters for each strain was determined using T-REx [19]. Genes within a *k*-means cluster are proposed to be close in a GCN; either through a direct or via an indirect connection (since correlation is a transitive measure). Analysis of the RNA-Seq data (*B. subtilis* 168 and *B. amyloliquefaciens*) and determination of DEGs was done using T-REx [19]. We use the term cluster to refer to the list of genes that show similar expression profiles as determined by the *k*-means algorithm. In contrast, the term module refers to a list of genes grouped together in the same part of a co-expression network. Essentially, modules are determined from the reconstructed co-expression network (often based on association measures), using module detection algorithms.

### Structural properties of the *B. subtilis* 168 and *B. amyloliquefaciens* sporulation networks

Biological networks are not randomly connected but rather organized into modular hierarchical structures that can be described using mathematical formulations. The truncated power-law distribution has been used to describe the degree distribution of biological [47] and social networks [48, 49]. The truncated power-law distribution is considered as a power-law distribution with the model formulated as $P\left(\eta \right)∼\eta^{-\gamma }$. The distribution has a sharp drop-off in higher degree nodes. It is expressed as

$$P\left(\eta \right)=\beta \eta^{-\gamma }exp\left(-\alpha \eta \right)$$

Here β and α are constants and γ is the power law exponent, $\eta_{i}=\sum_{j}a_{ij}$ is the total number of edges connected to node i. The probability that a node i in a network has $\eta_{i}$ edges is given by $P\left(\eta \right):=P(\eta_{i})=\eta_{i}/\sum_{j}\eta_{j}$ which is also the proportion of nodes with degree η [50]. The model was fitted to the degree distribution of the *B. subtilis* 168 and *B. amyloliquefaciens* networks. Additionally, we obtained the average clustering coefficients [51] for all nodes in each network. The clustering coefficient explains the extent of completeness of the neighbors of a particular node.

## Results

### The *B. subtilis* 168 GCN and sporulation-associated genes

**Fig. 1.**
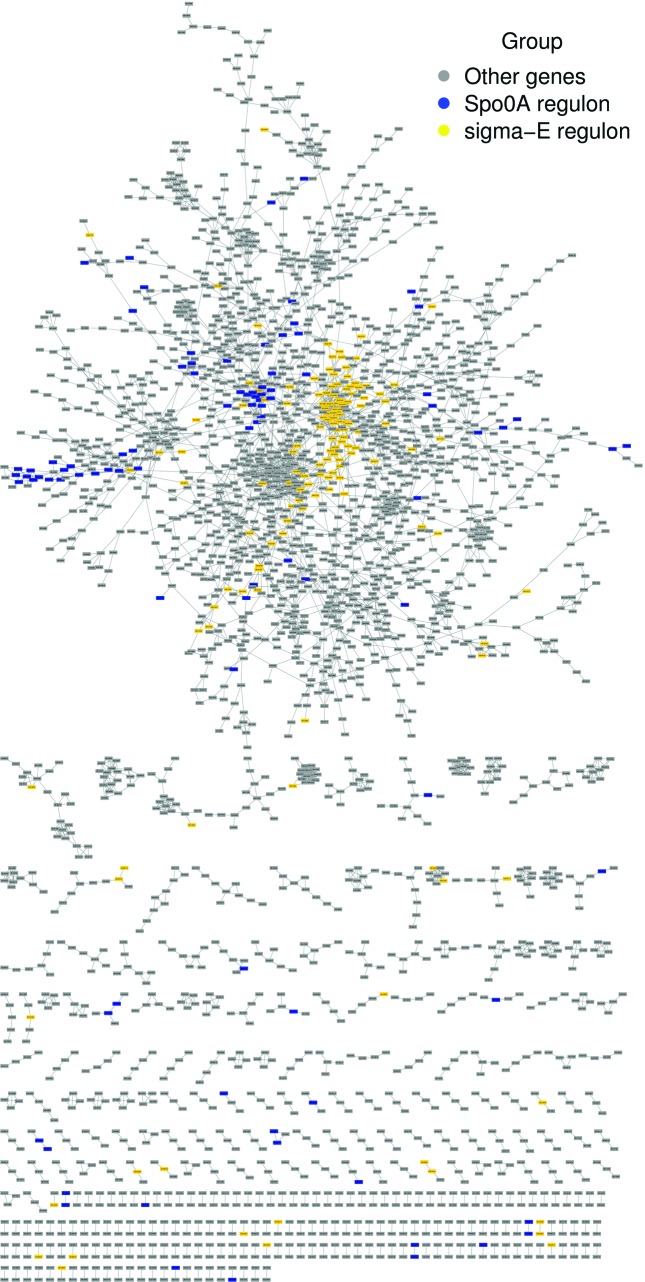
Bench-marking the *B. subtilis* 168 GCN generated using data from Nicholas *et al.* [23]. The sporulation network showing genes belonging to the Spo0A regulons (93 genes in blue) and σ^E^ regulons (144 genes in yellow); these genes met the filtering criterion for inclusion in the network generated using SPACE. Most genes in each of these two regulons cluster within the same vicinity in the network. Such a spatial distribution of genes can be expected since GCN is generated from transcriptome data while the benchmark gene lists for the regulons come from online databases.

Generally, network reconstruction involves identification of groups of genes with similar expression profiles across conditions or time. Genes that have correlated expression profiles to one or more other genes will be included in the GCN and those with highly similar expression profiles are grouped together in modules within the GCN. We discuss two interesting sets of genes, while bench-marking the network, namely: (i) the Spo0A and σ^E^ regulons (sigma-E, Fig. 1) and (ii) two gene clusters derived from DEG analysis (genes that were differentially expressed across the six conditions; Fig. S1). A selection of clusters with distinct profiles in which most genes showed a similar expression pattern across conditions was used to bench-mark DEGs obtained from *B. subtilis* 168 RNA-Seq data. DEGs from both strains were mapped onto the co-expression network to visualize the distribution of gene classes (Fig. S1).

In the analysis, hubs were defined as genes connected to at least five other genes in the same network [52, 53]. The GCN depicted in Fig. S1 has a high modularity with *Q*≈0.89 and contains 832 hubs (File S4). Gene set enrichment analysis (GSEA) of the modules of this network (Table S1) showed enrichments associated with sporulation, such as module 9 (GO:0030435, sporulation resulting in formation of cellular spore), module 13 (GO:0030436, asexual sporulation) and module 80 (GO:0009847, spore germination) (all with *P*<0.01). Although not discussed here, many modules in the GCN (Table S1) showed enrichment of various biological processes. As discussed later, the *B. amyloliquefaciens* network is highly modular, *Q*=0.85 (Fig. 5a) indicating that most genes in the GCN are grouped into modules (as shown in Fig. S2) associated with specific biological functions. A functional analysis [19] of the genes in cluster 5 (determined using *k*-means) showed that the top hits for regulons are a regulon of the stage V sporulation protein SpoVT (*P*=1.19e−8) and a regulon of the anti-terminator protein, GlcT (*P*=2.48e−2). Additionally, the Gene Ontology (GO), Kyoto Encyclopedia of Genes and Genomes (KEGG) pathway and cluster of orthologous groups (COG) analysis all showed genes (from cluster 5) significantly associated with spore germination, spore wall, the spore coat and spore formation (Table S2), essentially constituting a list of genes important for spore germination. The sporulation auxiliary transcriptional regulator GerE (*P*=1.57e−11) was the only top hit in cluster 8 (8 out of 20, top hits by class size; Table S3). GerE activates or represses genes linked to spore coat formation [54].

### The Spo0A and σ^E^ sporulation sub-network in *B. subtilis* 168

Starting from the global network, which consists of 2867 genes and 4436 edges (Fig. S1), sub-networks of the Spo0A (93 genes, 108 edges) [55, 56] and σ^E^ (144 genes, 229 edges) [57] were generated using sporulation gene-lists from SporeWeb. The combined sub-network of only the Spo0A and σ^E^ regulons had 237 nodes and 337 edges. Genes with a first-degree (a direct) connection to the mapped nodes were also extracted, resulting in a network of 500 genes and 896 edges (for both the Spo0A and σ^E^ regulons). On the basis of the results of the guilt-by-association analysis, 263 genes in the sub-network (Fig. 4a) were linked to sporulation as members of at least one of these two regulons. This includes the genes that were already identified to belong to the Spo0A and σ^E^ regulons.

A gene set enrichment analysis of these 263 genes showed that indeed genes involved in sporulation are directly connected to the Spo0A and σ^E^ regulons in the sub-network. The genes *yabS–yabT*, encoding a protein with unknown function and a serine/threonine kinase, respectively, are predicted (by Genome2D [58]) to be in an operon with *spoIIE* and are connected to the sub-network. This has also been corroborated in a previous work [59] in which *spoIIE* and *yabST* have been established to be transcribed together and, therefore, to have a similar regulation mechanism. Although the *spsABCDEFGIL* spore coat polysaccharide biosynthesis operon and the spore germination operon *gerP-ACDE* are not known to be under control of Spo0A and/or σ^E^, the reconstructed GCN shows a direct connection to these regulators, although the modes of their specific regulation mechanisms remain unclear. Therefore, these operons are interesting candidates for future studies on genes involved in sporulation. Furthermore, some of the 263 genes belonged to some operons encoding mainly hypothetical proteins, operons with gene expression profiles that are similar to those of the genes in the Spo0A and σ^E^ regulons (Fig. 4a).

To further assess the network enrichment, regulons and genes involved in sporulation were mapped onto the *B. subtilis* GCN. This mapping indicated that genes of the Spo0A and σ^E^ regulons were clustered closely in the network (Fig. 1). Spo0A governs transcription at the onset of sporulation [56, 60] and σ^E^ is a mother-cell-specific σ factor that directs gene transcription in the mother cell after asymmetric sporulation cell division [57, 61].

**Fig. 2.**
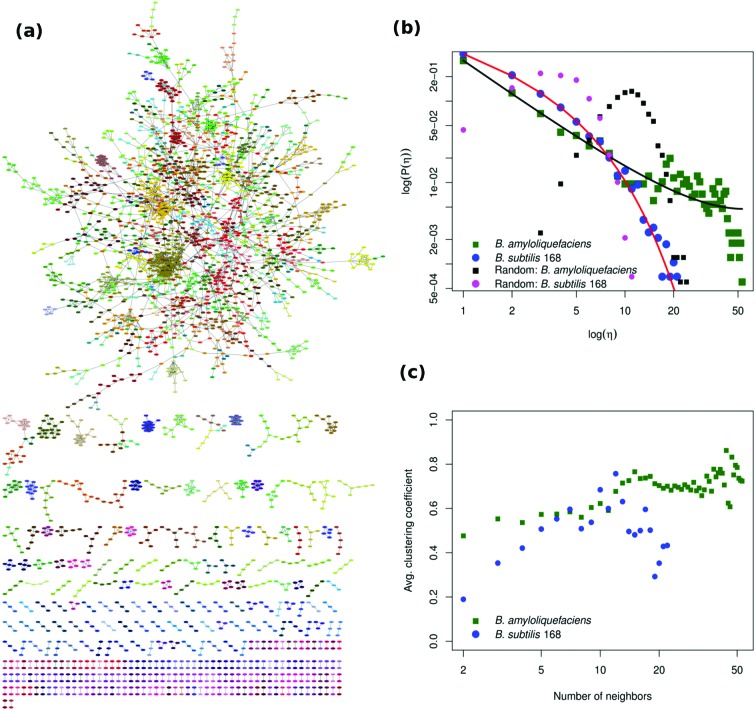
Detected modules in the *B. subtilis* 168 and *B. amyloliquefaciens* GCN and their structural properties. (a) The network has 2867 genes (nodes) and 4436 edges (black lines connecting nodes). The 611 modules are indicated in different colors. A threshold of ρ=0.35 was used for the partial correlation measure during network reconstruction using SPACE. The network was visualized using Cytoscape v3.2.1. The nodes shown in different colors indicate gene membership of the network modules and the number of genes in the individual modules varies widely. (b) Truncated power law distribution plot for the *B. subtilis* 168 and *B. amyloliquefaciens* GCNs, plot on a log–log scale. The model fits through the data points show significantly good fits. This means that the model provides a good description of the degree distribution of the network nodes. These plots show that many nodes have few connections while a few nodes are highly connected. Included are plots of the degree distribution of randomly connected networks with the same size and number of edges as that of the *B. subtilis* 168 and *B. amyloliquefaciens* GCN. The degree distribution of the networks for the two strains is shown in black and magenta. (c) Distribution of the corresponding average clustering coefficients for the two networks.

The truncated power-law distributed networks, which exhibit a scale-free behavior, have significant roles in biology since they typically have hubs organized in modules [62] and the number of connections per node is a crucial measure of network topology. To investigate the topological properties of the *B. subtilis* 168 GCN (Fig. 2a), we fitted a truncated power-law distribution model to the degree distribution of its nodes and compared it with that of the *B. amyloliquefaciens* network (Fig. 2b). The degree distributions of the *B. subtilis* 168 and *B. amyloliquefaciens* GCN differ from those of randomly connected networks with the same sizes and edges (Fig. 2b), meaning that the two networks are hierarchically organized in modular structures. Unlike networks with randomly connected nodes, the connections in a GCN generally follow specific distributions, they are robust and any loss of non-hub nodes is non-lethal. Our analysis showed that about 12.5 % of the nodes in the *B. subtilis* 168 GCN were paired and detached connected nodes (e.g., 358 nodes out of 2867 identified nodes). Additionally, in the *B. subtilis* 168 GCN, 105 nodes which are typical of hubs and transcription/sigma factors were connected to over 20 other nodes (Fig. 2b). This is consistent with the expected behavior of the *B. subtilis* 168 network, which has multiple sigma factors and auxiliary transcriptional regulators that regulate the expression of sporulation genes. In Fig. 2(c), most nodes have a clustering coefficient averaging 5 to 15 neighbors, which indicates the close association between genes within the modules. The genes *adhB* (forespore-specific protein) and *yhcA* (similar to multi-drug resistance) [31] had the highest connectivity in the network, each having 39 targets. Both genes have unknown functions.

### Variations in DEGs between *B. subtilis* 168 and *B. amyloliquefaciens* during sporulation

**Fig. 3.**
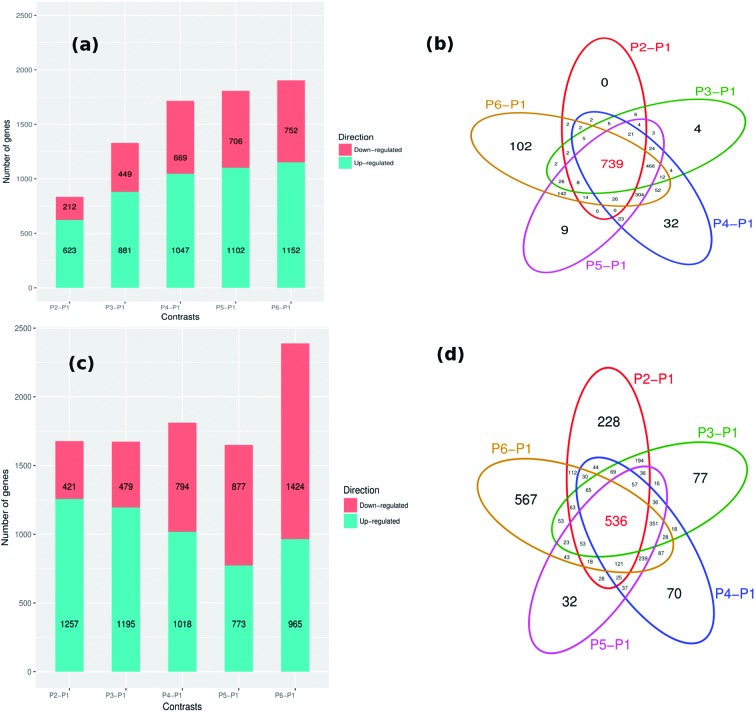
Time progression of sporulation for the *B. subtilis* 168 and *B. amyloliquefaciens* networks. (a) and (c): Bar plots of the number of DEGs at the various stages of sporulation, time instants P2 to P6 all referenced to P1 (i.e., P2–P1 to P6–P1, see Methods). (b) and (d): Corresponding Venn diagrams indicating the overlap for the subsets of the number of DEGs found at the time contrasts (P2–P1 to P6–P1) for (b) *B. subtilis* 168 and (d) *B. amyloliquefaciens*, respectively. The number of DEGs is indicated in each sector of the Venn diagram. The colored ellipses in indicate the different contrasts for the Venn diagram.

The time-progression of sporulation was explored by projecting differentially expressed genes at P2 to P6 (with reference to P1, see Methods). Interestingly, by assigning the differentially expressed genes a different color compared with the other genes in the network, and subsequently plotting them, we found that different parts of the network are highlighted during the progression of sporulation. This is attributed to differential expression of a varying number of genes over time (looking at *B. subtilis* 168, Fig. 3a), which indicates the influence of transcription factors that are turned off and on at specific stages of sporulation. For *B. subtilis* 168, it is clear that the number of both down-regulated and up-regulated DEGs increased with time (Fig. 3a) although such an increase is not apparent for *B. amyloliquefaciens* (Fig. 3b). Overall, 739 DEGs were common to all the time instants (for *B. subtilis* 168, Fig. 3b), and all genes that were differentially expressed at P2 were found to be differentially expressed in at least one of the subsequent stages (P3 to P6). Few DEGs were observed only within the contrast P2–P1 (no genes) and P3–P1 (4 genes), Fig. 3(b).

The DEGs were also determined for *B. amyloliquefaciens* using T-REx [19]. An increasing number of DEGs was found ranging from the initial to the late stages of sporulation (Fig. 3c). Like the trend for *B. subtilis* 168 (Fig. 3a), most DEGs in *B. amyloliquefaciens* were found at P6. In contrast to the time-evolution trend for DEGs in *B. subtilis* 168 (Fig. 3a), the profiles for *B. amyloliquefaciens* show a steadily decreasing number of up-regulated genes (except at P6–P1, Fig. 3c) and an increasing number of down-regulated genes. The number of significantly up-regulated genes for *B. subtilis* 168 outweighs that for down-regulated genes at the same stages of sporulation (Fig. 3a). However, *B. amyloliquefaciens* has an opposite profile particularly at the later stages of sporulation (Fig. 3c). Unlike *B. subtilis* 168, which had no DEGs in P2–P1 (Fig. 3b), in *B. amyloliquefaciens* a total of 228 genes were uniquely differentially expressed at P2–P1 (Fig. 3d). Remarkably, a large number of DEGs (567) were unique to the last sporulation stage (P6–P1), Fig. 3(d). Overall, 39–56 % of the genes in the *B. amyloliquefaciens* genome were significantly differentially expressed compared with 20–45 % in *B. subtilis* 168. This deviation could explain the ability of *B. amyloliquefaciens* spores to survive harsher environmental conditions than *B. subtilis* 168 spores [63].

### Enriched functional modules in *B. subtilis* 168 and *B. amyloliquefaciens* GCNs

**Fig. 4.**
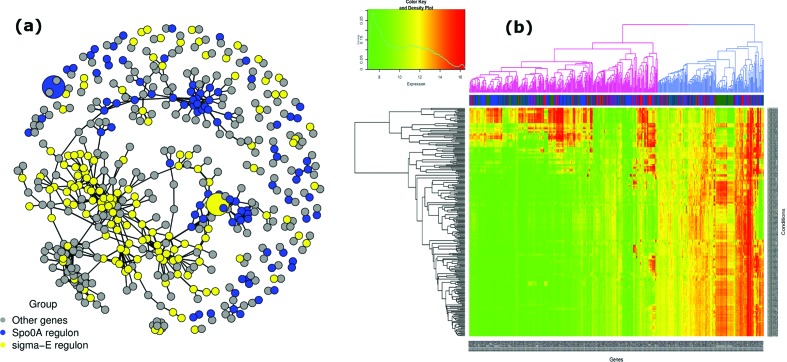
Sporulation sub-network of *B. subtilis* 168. (a) Sub-network of the Spo0A and σ^E^ regulons with a first-degree connection to the ‘Other genes’. The co-expression network consists of 500 genes and 896 edges. Edge thickness represents the strength of association between nodes; the thicker the edge, the stronger the association. The larger blue and yellow nodes are the *spo0A* and *gerE* genes which encode Spo0A and σ^E^, respectively. (b) Bi-directional clustering of the 500 genes and 255 conditions. The genes generally group in two broad clusters (indicated in purple and blue). Most genes from the first (purple) cluster have on average low expression, while those from the second (blue) cluster have on average higher expression, as seen from the figure color key and density plot. The Euclidean distance was used throughout for the distance calculations.

To explore the intrinsic intermediate regulatory effects, a sub-network was extracted from the *B. subtilis* GCN as shown in Fig. 4(a). This sub-network had 500 genes, 237 of which belonged to the Spo0A and σ^E^ regulons. All the genes associated with these regulons (93 for the Spo0A regulon and 144 for the σ^E^ regulon) and 263 genes directly connected to these regulons were subjected to gene set enrichment analysis. The function enrichments for the network modules (Table S4) revealed significant protein family (InterPro, IPR) classifications for spore coat protein CotH, involved in spore coat assembly [64] (adjusted *P* = 0.0028) and spore germination protein family GerPA–GerPF [65] (adjusted *P* = 0.0127), analysis done using T-REx. The operon encoding GerPA–GerPF proteins belongs to regulons consisting of the two sporulation-related transcriptional regulators, σ^K^ and GerE [57]. The *gerPA–gerPF* operons consist of six genes that encode the GerPA, GerPB, GerPC, GerPD, GerPE and GerPF proteins. As mentioned above, the *gerP* operon is part of the SigK regulon [65]; therefore, it is conceivable that expression of its genes is also affected by Spo0A and SigE. There is also likely to be some level of cross-talk between the network components (genes), a phenomenon that is not uncommon in co-expression networks [66]. Similarly, expression of *cotH* is regulated by the two sequentially acting mother-cell-specific sporulation σ factors, σ^E^ and σ^K^ [61]. Additionally, *cotH* is under the positive control of the sporulation-specific secondary transcriptional regulator SpoIIID. A further functional analysis results in top hit regulons: YrxA (later named NadR in literature, *P* = 0.0003), SpoVT (*P* = 0.0014) and GerE (*P* = 0.0026). All these regulons have been previously linked to some stage of sporulation in bacteria [67]. A number of significant GO groups and cluster of orthologous genes (COGs) with single entries were also detected in the gene set enrichment analysis (Tables S2 and S3). This is possible because only single genes were annotated in these GO classes. Further details on the COG, GO terms, InterPro, KEGG and operons of those 263 genes are found in Table S4.

The expression profiles of the 500 genes (including those in the Spo0A and σ^E^ regulons) and 255 conditions were clustered using the hclust function for hierarchical clustering in R software and visualized in a heatmap (Fig. 4b). This figure shows how, for instance, the expression profiles of genes directly associated with the Spo0A and σ^E^ regulons map on the literature-based information. Fig. 4 has three main gene classes (Spo0A, σ^E^ regulons and the other genes). There is no clear-cut one-to-one association between the two gene clusters in violet and blue; however, many genes cluster together in large numbers (big blue and dark-green column color bands). These large color bands correspond to the densely grouped genes in the sub-network (Fig. 4a, b).

A modularity value *Q≈*1 indicates the presence of more edges within the modules than would be expected by chance, while *Q≈*0 indicates a randomly connected network [43]. In our case, the *B. subtilis* 168 GCN is organized in a highly modular structure (2867 genes, 4436 edges). The 2867 genes represent 65 % of the genes in the genome (with a total of 4230 genes). The other 35 % of the genes in the genome were filtered out during the network reconstruction using SPACE. We also performed gene set enrichment analysis on the *B. amyloliquefaciens* GCN and show that the network modules are enriched with various functions (Table S5). This table provides an overview of the significantly enriched GO terms in the different network modules, e.g., module 11 (Table S5) specifically being enriched to for spore germination.

### Enrichment analysis of genes highly expressed at all time-points for *B. subtilis* 168 and *B. amyloliquefaciens*

By analyzing the expression profiles for all genes in time, we ranked all the genes in each genome based on their minimum expression value at all the time instants. We extracted the top 10 % most expressed genes in both the *B. subtilis* 168 and *B. amyloliquefaciens* RNA-seq data. The list of genes for each strain was then subjected to downstream gene set enrichment analysis in Genome2D [58]. The gene set enrichment results for the analysis for the top 10 % most expressed genes across time points for *B. subtilis* 168 and *B. amyloliquefaciens* are given in Tables S6 and S7, respectively. From these tables, we observed that the top most enriched biological processes are classified by GO terms as the structural constituents of ribosomes, intracellular, ribosome and translation (adjusted *P* values <1.0e−27) for *B. subtilis* 168, and structural constituents of ribosomes, intracellular, ribosome and translation (adjusted *P* values <1.0e−44) for *B. amyloliquefaciens*. For both strains, some significantly enriched GO terms are associated with: metabolic process, (glucose, cellular amino acid, carboxylic acid), ATP-binding cassette (ABC) transporter complex, (ATP synthesis, ATP hydrolysis) coupled proton transport and ATP synthesis-coupled electron transport (for *B. subtilis* 168), and cellular amino acid metabolic process, ATP metabolic process, glucose metabolic process and ATP binding (*B. amyloliquefaciens*). Overall, there is a large spectrum of processes that are significantly enriched. Remarkably, some of the top enriched GO terms include translation, intracellular, ribosome, RNA binding and structural constituent of ribosome (all with adjusted *P* values <1.0e−14, Tables S6 and S7, for both strains).

### *B. amyloliquefaciens* GCN has a similar structure to that of *B. subtilis* 168 but differs in biological enrichment

**Fig. 5.**
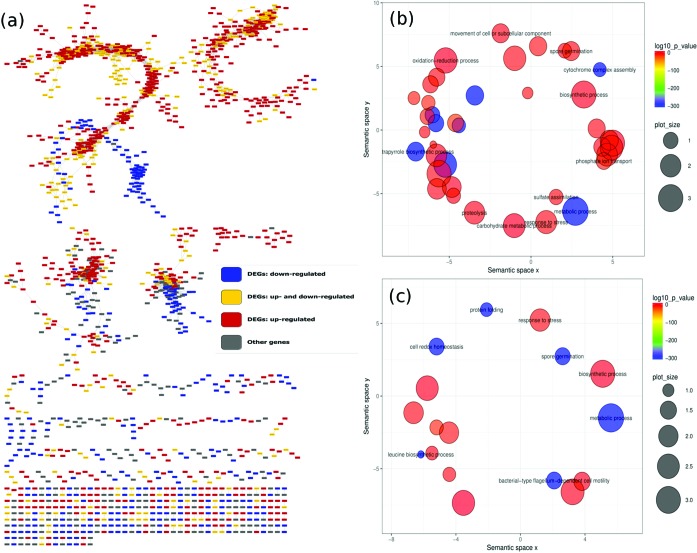
*B. amyloliquefaciens* gene co-expression network (1665 nodes, 8287 edges). (a) DEGs that were consistently up-regulated and down-regulated under all conditions are colored red and blue. The genes that were both differentially up-regulated and down-regulated across the time contrasts (P2 to P6) are shown in yellow nodes. The gray nodes are the non-DEGs. (b) The Reduce Visualize Gene Ontology (REVIGO) [68] projection of all enriched GO terms from the *B. subtilis* 168 GCN modules. (c) Equivalent projection of all modules from *B. amyloliquefaciens* GCN. The significance of the enrichment test is represented by the color intensity. A semantic depiction of the GO categories colored according to significant over-representation in the GCN modules. The GO terms in the blue and green bubble circles are more significantly enriched, lower log_10_(*P*-values) than those in orange and red (legends in upper right hand corner of the respective plots). The sizes of the GO terms are indicated by the circle radiuses, the larger the circle, the more over-represented the GO term. The semantic spaces *x* and *y* correspond to the multidimensional scaling of the matrices of the GO terms' semantic similarities.

Like *B. subtilis* 168, the *B. amyloliquefaciens* network has a scale-free distribution (Fig. 2b), which is typically observed for most realistic gene networks [50] and the nodes in the networks are hierarchically organized (Fig. 5a). Even though both GCNs are scale-free, the latter is more densely connected and has larger sized-modules (Fig. 2b, c). Both networks exhibit variations in enrichments within their modules. The variations in enrichment and number of DEGs at the same time-instants (Fig. 3) explain the differences in the sporulation network between the strains.

Hubs were extracted from this network and the detected modules were subjected to gene set enrichment analysis. File S5 contains a list of hub genes from the *B. amyloliquefaciens* GCN. The gene set enrichment analysis results for modules with significantly enriched gene sets are given in Table S5. This table shows various interesting examples of modules containing genes related to sporulation, e.g. in module 11 (GO:0009847) with genes involved in spore germination. To compare the enrichment in the *B. subtilis* 168 and *B. amyloliquefaciens* GCNs, we projected all significantly enriched GO terms onto the webserver REVIGO [68], which is a tool used to summarize and visualize long lists of GO terms. These projections represent significantly enriched biological processes from the GO enrichment of gene sets from the *B. subtilis* 168 and *B. amyloliquefaciens* network modules using REVIGO [68]. It also enables searches for a representative subset of terms, visualization of non-redundant GO terms and performing of multidimensional scaling and graph-based visualizations. Some GO enrichments terms corresponding to metabolic processes and biosynthesis processes are well represented in both GCNs. However, spore germination is more enriched in the *B. amyloliquefaciens* network (Fig. 5c) than in the *B. subtilis* 168 network (Fig. 5b). This could lead to improved understanding of the germination and sporulation properties between the two strains, which would be interesting for further studies.

### Comparison of conserved modules in both the *B. subtilis* 168 and *B. amyloliquefaciens* GCNs

To assess the level of similarity between the *B. subtilis* 168 and *B. amyloliquefaciens* networks, we integrated the two GCNs to determine common nodes and edges between them (Fig. S3, resulting in 1102 nodes and 90 edges). The intersection of the two networks has conserved modules with specific and in some cases generic functions as depicted in the enriched GO terms (File S3). Two modules are specifically conserved for the formation of cellular spore and spore walls (module 2 and module 3, Table S8), while the other modules represent more generic biological functions (e.g., transferase and catabolic activity, metabolic processes, etc., in various modules, namely: module 1, module 4, module 5, module 6 and module 7, Table S8). This result indicates that there are certain modules with specific node connections that are commonly maintained between the networks of the two strains (*B. subtilis* 168 and *B. amyloliquefaciens*).

## Discussion

We used high-resolution transcriptome data to generate and analyze sporulation GCNs for *B. subtilis* 168 and *B. amyloliquefaciens*. Our results illustrate the power of GCNs and hierarchical data clustering in determining enriched network modules. Such gene clusters supplement the list of top candidate genes considered to be involved in specific mechanisms, e.g. in our study sporulation. A comparison of the *B. amyloliquefaciens* network with that of *B. subtilis* 168 was performed, because it is a well-studied strain for which a lot is known concerning transcriptomics, sporulation gene expression/regulatory network and transcriptional regulators. Because of this richness of already available data, it was chosen as a reference strain. The differences in *sigH* expression in *B. subtilis* 168 would rather influence the transition state (fate decision-making process)/sporulation initiation and would have little influence on the stages of sporulation after the asymmetric division. Even lower level of *sigH* activity should not influence the connections between the genes; our network mainly focuses on the network connections and how the genes associate with each other, rather than assessing the effects of the expression levels of individual genes on the network dynamics.

In *B. subtilis* 168, various genes are regulated at specific stages of sporulation; therefore, such groups of genes were deduced from GCN. In our network, transcription factors and their targets did not always appear in the same module because GCNs are generated using similarity in gene expression patterns, and transcription of many genes is controlled by multiple transcription factors. It is even more complicated to detect regulatory mechanisms for transcription factors that are both activators and repressors, e.g., the sporulation-specific transcriptional factors, SpoIIID [69], GerR [67, 70] and GerE [67]. We show that targets of these transcription factors have at least two distinct expression profiles across conditions; hence, the target genes appear to be spatially distributed in different network modules to their transcription factors, especially genes under the negative regulation of the transcription factor. The modules in the GCNs are connected by genes such as *sigE* which belongs to both the σ^E^ and Spo0A regulons (also corroborated by SubtiWiki). The expression of *spo0A* is under the control of its own phosphorylated product (Spo0A~P), sigma factors SigA and SigH and the transcription factor SinR. Such regulatory mechanisms are difficult to discern from transcriptome data, irrespective of the robustness of the network reconstruction method. This explains why *sigE* and especially *spo0A* appear partially detached from the other genes in the GCN. Moreover, the activity of Spo0A is regulated at the post-transcriptional level through its phosphorylation. Different concentrations of Spo0A~P in the cell turn genes on and off. Additionally, the activity of other sporulation-specific regulators is controlled at the post-transcriptional level.

*sigH* controls transcription of early stationary phase genes [31]. The lower number of DEGs at P2–P1 in *B. subtilis* 168 may be caused by the V117A *sigH* mutation that has been recently described as emerging in domesticated strains of *B. subtilis*, including 168 [71]. The mutation has been suggested to decrease the rate of accumulation of phosphorylated Spo0A and affect the rates of expression of early sporulation genes [71]; however, this does not rule out other possible causes of the lower number of DEGs compared with the subsequent stages of sporulation. *B. subtilis* cells entering the stationary phase in the sporulation cycle are faced with numerous decisions. At this stage, the highly interconnected regulatory network components control differential gene expression. Such regulatory circuits direct the cell along the most favorable survival path, subject to the environment in which the cell is located [72].

We analyzed the structural properties of the networks, assessed the network modules for their biological function enrichment and compared the differential gene expression and time-progression of sporulation in both strains. Our work provides leads for candidate genes for future studies, e.g. for identification of potential pathways and biomarker genes involved in various processes in the cell. It gives an unprecedented look at the dynamics of gene regulation processes during all phases of sporulation. Although reconstruction of complete and fully reliable genome-scale co-expression network remains a challenge, we have demonstrated that interesting results can be obtained using high-quality transcriptome data and robust gene network inference, thereby improving our understanding of sporulation in *B. subtilis* 168 and *B. amyloliquefaciens* and fuelling further leads for research on clusters of genes of specific interest.

The sporulation network presented here can be mined for genes of interest for future studies, e.g., *yrbC* and *yabS*, whose role in sporulation remains poorly understood, even though *yrbC* has previously been linked to sporulation [73], and the closely associated modules to which they belong. Such leads for new investigations can be obtained through inspection of genes of interest within a network module, especially the genes that are highly connected [23, 74]. Without GCNs, it is very challenging to identify candidate genes considered to be either co-regulated or associated directed or indirectly in their regulation mechanism. Such analysis enables us to group genes involved in the same ontology (i.e., biological process, molecular function, or cellular component). Some of these factors are sporulation-stage-dependent and might vary between strains. The sporulation networks show enriched modules with genes belonging to regulons that are associated with specific stages of sporulation and other non-sporulation-related processes. Although reconstruction of GCNs on the basis of high-throughput transcriptomic data still falls short of making highly reliable predictions of sporulation transcriptional behavior, comparison and mapping of conserved network modules enable identification of candidate genes involved in sporulation and those that are associated with specific sporulation stages. Altogether, the GCNs serve to extend our understanding of sporulation in Bacilli and they also provide a platform for analyzing additional closely related strains.

## Data bibliography

1. All supporting data is available in the online version of this article.

## Supplementary Data

1309 Supplementary File 1
