# Supplementary material for: Dynamic sporulation gene co-expression networks for Bacillus subtilis 168 and the food-borne isolate Bacillus amyloliquefaciens: a transcriptomic model
Source: Microb Genom. 2018 Feb 9;4(2):e000157. doi: 10.1099/mgen.0.000157 (PMC5857382; doi:10.1099/mgen.0.000157)
Supplement: Supplementary File 1 [file mgen-4-157-s001.pdf]

Supplementary material

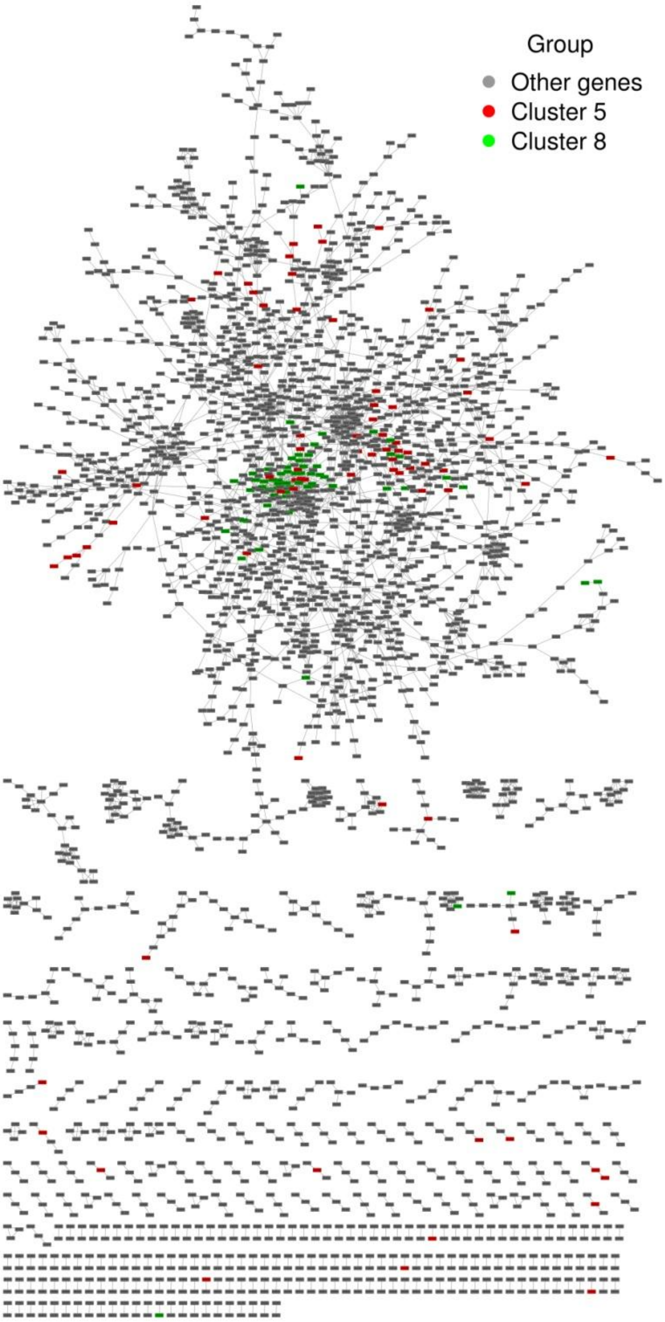

Fig. S1. Further bench-marking the *B. subtilis* 168 GCN generated using data from (1). Cluster 5 (77 genes) and cluster 8 (60 genes) derived from the *k*-means clustering of differentially expressed genes are highlighted in red and green, respectively. Most genes in these two clusters are located within the same vicinity in the network. Figure also available at URL: https://figshare.com/s/db10961af7c2b20d6a23, DOI: 10.6084/m9.figshare.5099941.

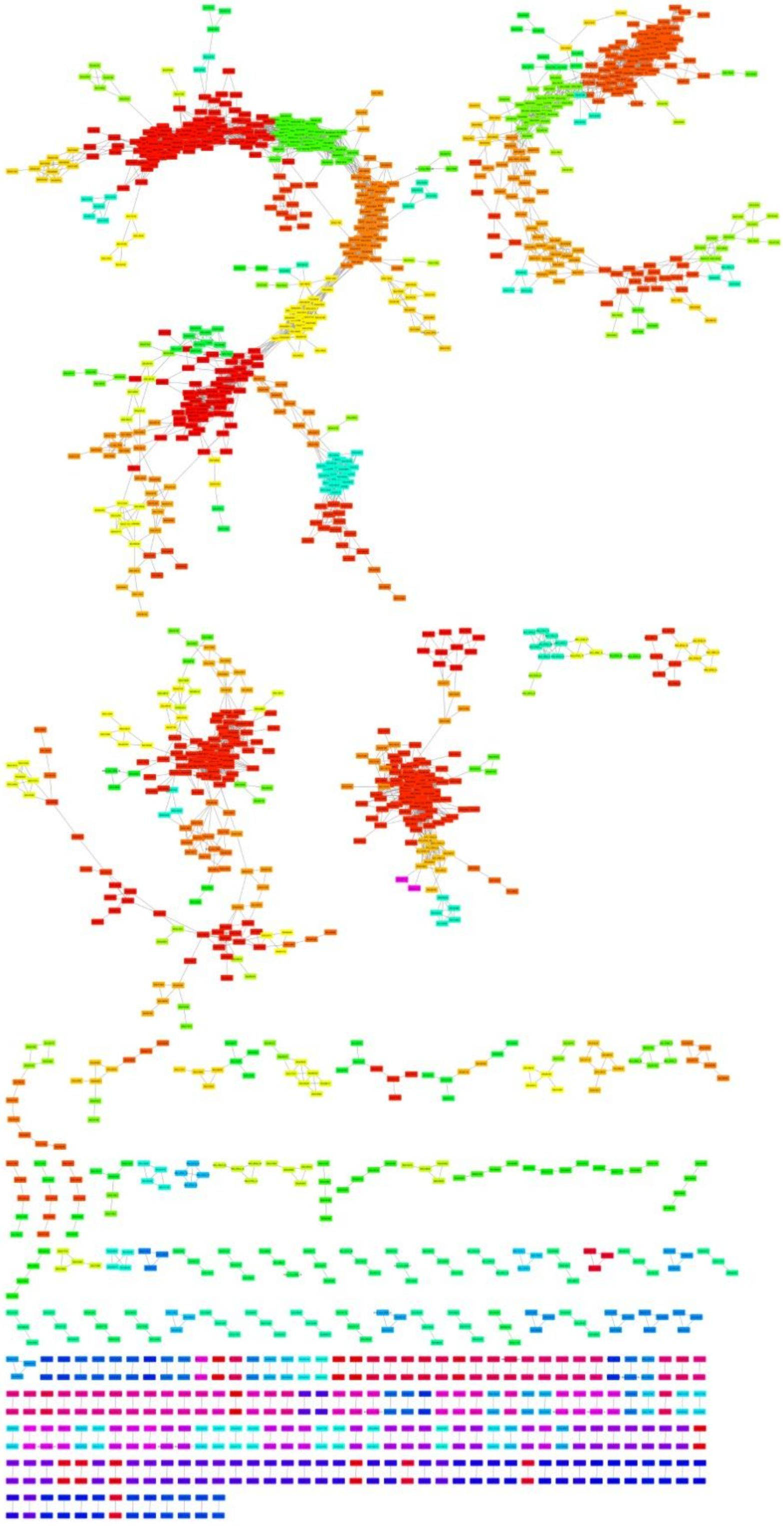

Fig. S2. ***B. amyloliquefaciens*** **GCN.** Network of 1665 nodes and 8287 edges. The different colors indicate the 361 detected modules. Figure also available at URL: https://figshare.com/s/964bb2ea72f74f063e36, DOI: 10.6084/m9.figshare.5099944.

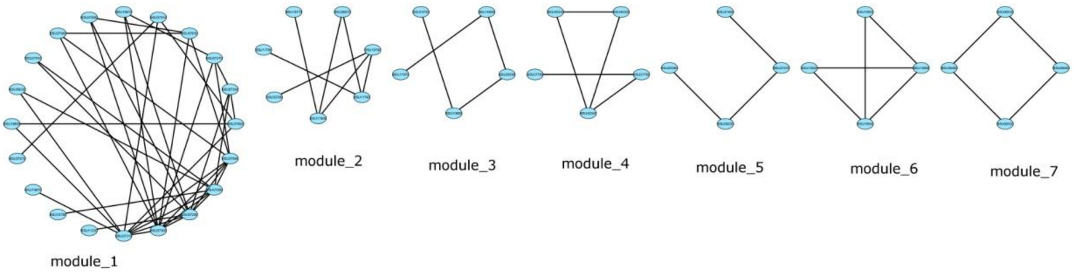

Fig. S3. Conserved modules in both the *B. subtilis* 168 and *B. amyloliquefaciens* GCNs (determined using Cytoscape). Part of the network of 1102 nodes and 90 edges resulting from intersecting both GCNs. Enrichments within each module are in Table S8*.* Only conserved modules with size ≥4 are presented. Figure also available at URL: https://figshare.com/s/46d20371e4b9c8a379aa, DOI: 10.6084/m9.figshare.5314576.

Table S1. Gene set enrichment for all modules from the *B. subtilis* 168 gene co-expression network. Only significantly enriched modules are presented. URL: https://figshare.com/s/0010bada2512f558d6cc, DOI: 10.6084/m9.figshare.5099935.

Table S2. Cluster 5: Gene Ontology (GO). URL: https://figshare.com/s/f03664f1ea47e26c2bae, DOI: 10.6084/m9.figshare.5099923.

Table S3. Cluster 8. Gene Ontology (GO). URL: https://figshare.com/s/45d63ceb63a150f1e5c9, DOI: 10.6084/m9.figshare.5099926.

Table S4. Results from the GSEA from the genes associated to sporulation. The analysis is based on genes directly connected to the genes in the Spo0A and σ^E regulons in the *B. subtilis* 168 sub-network. URL: https://figshare.com/s/f65189e65e3ee6bd15ac, DOI: 10.6084/m9.figshare.5099929.

Table S5. Gene set enrichment for all modules from the *B. amyloliquefaciens* co-expression network. Only significantly enriched modules are presented. URL: https://figshare.com/s/0d0b1d96c6226242ca8d, DOI: 10.6084/m9.figshare.5099938.

Table S6: *B. subtilis* 168, enrichment of genes highly expressed across all time-points. Results of gene set enrichment analysis for prokaryotes (GSEA-Pro). Cutoff value for the adjusted p-values is 0.01. The number of genes, per class, that

are differentially expressed (TopHits) are indicated in brackets. The rating values (1 to 5) reflect binned values based on: (TopHits/ClassSize)*-$\log_2$(adj-pvalue). URL: https://figshare.com/s/8c855986d03f663ea95f, DOI: 10.6084/m9.figshare.5634292.

Table S7: *B. amyloliquefaciens*, enrichment of genes highly expressed across all time-points. Results of gene set enrichment analysis for prokaryotes (GSEA-Pro). Cutoff value for the adjusted p-values is 0.01. The number of genes, per class, that

are differentially expressed (TopHits) are indicated in brackets. The rating values (1 to 5) reflect binned values based on: (TopHits/ClassSize)*-$\log_2$(adj-pvalue). URL: https://figshare.com/s/5a66268956490083 72d8, DOI: 10.6084/m9.figshare.5634325.

Table S8. Overrepresented classes of GO terms in conserved network modules from the *B. subtilis* 168 and *B. amyloliquefaciens*. The class names contain links to internet resources, hits are the number of genes from the 'top-list' in a class. The p-value is the summation of the hyper-geometrical distribution calculated for each class. Only conserved modules with size ≥4 are presented. Table available for download from URL: https://figshare.com/s/bc6ff3c7d631f7826a19, DOI: 10.6084/m9.figshare.5314516.

Module_1

**CLASS**	**Top hits in Class**	**Class size**	***p*-value**
GO:0016757 transferase activity, transferring glycosyl groups	**3**	31	3.18e-04
GO:0003824 catalytic activity	**3**	54	1.64e-03
GO:0050662 coenzyme binding	**2**	20	3.45e-03
GO:0009058 biosynthetic process	**2**	22	4.17e-03
GO:0008460 dTDP-glucose 4,6-dehydratase activity	**1**	1	4.49e-03
GO:0008830 dTDP-4-dehydrorhamnose 3,5-epimerase activity	**1**	1	4.49e-03
GO:0008831 dTDP-4-dehydrorhamnose reductase activity	**1**	1	4.49e-03
GO:0009225 nucleotide-sugar metabolic process	**1**	1	4.49e-03
GO:0045226 extracellular polysaccharide biosynthetic process	**1**	1	4.49e-03
GO:0047343 glucose-1-phosphate cytidylyltransferase activity	**1**	1	4.49e-03
GO:0015105 arsenite transmembrane transporter activity	**1**	2	8.95e-03
GO:0016051 carbohydrate biosynthetic process	**1**	2	8.95e-03
GO:0016779 nucleotidyltransferase activity	**1**	4	1.78e-02
GO:0044237 cellular metabolic process	**1**	4	1.78e-02
GO:0046685 response to arsenic-containing substance	**1**	5	2.22e-02
GO:0009103 lipopolysaccharide biosynthetic process	**1**	7	3.10e-02
GO:0015774 polysaccharide transport	**1**	8	3.54e-02

Module_2

CLASS	Top hits in Class	Class size	*p*-value
GO:0030435 sporulation resulting in formation of a cellular spore	**4**	266	4.58e-04
GO:0031160 spore wall	**2**	35	1.36e-03

Module_3

CLASS	Top hits in Class	Class size	*p*-value
GO:0030435 sporulation resulting in formation of a cellular spore	**4**	266	7.23e-05

Module_4

CLASS	Top hits in Class	Class size	*p*-value
GO:0006525 arginine metabolic process	**3**	6	1.58e-08
GO:0006561 proline biosynthetic process	**2**	11	6.11e-05
GO:0004053 arginase activity	**1**	1	1.18e-03
GO:0004587 ornithine-oxo-acid transaminase activity	**1**	1	1.18e-03
GO:0003842 1-pyrroline-5-carboxylate dehydrogenase activity	**1**	2	2.36e-03
GO:0004352 glutamate dehydrogenase activity	**1**	2	2.36e-03
GO:0006520 cellular amino acid metabolic process	**1**	8	9.41e-03
GO:0015171 amino acid transmembrane transporter activity	**1**	24	2.80e-02

Module_5

CLASS	Top hits in Class	Class size	*p*-value
GO:0006468 protein phosphorylation	**1**	17	1.60e-02
GO:0017111 nucleoside-triphosphatase activity	**1**	29	2.71e-0

Module_6

CLASS	Top hits in Class	Class size	*p*-value
GO:0006207 'de novo' pyrimidine base biosynthetic process	**2**	4	4.01e-06
GO:0006221 pyrimidine nucleotide biosynthetic process	**2**	8	1.87e-05
GO:0004158 dihydroorotate oxidase activity	**1**	1	9.44e-04
GO:0004588 orotate phosphoribosyltransferase activity	**1**	1	9.44e-04
GO:0006222 UMP biosynthetic process	**1**	1	9.44e-04
GO:0044205 'de novo' UMP biosynthetic process	**1**	1	9.44e-04
GO:0004590 orotidine-5'-phosphate decarboxylase activity	**1**	2	1.89e-03
GO:0004088 carbamoyl-phosphate synthase (glutamine-hydrolyzing) activity	**1**	4	3.77e-03
GO:0009116 nucleoside metabolic process	**1**	9	8.47e-03

Module_7

CLASS	Top hits in Class	Class size	*p*-value
GO:0009113 purine base biosynthetic process	**2**	2	6.69e-07
GO:0006164 purine nucleotide biosynthetic process	**2**	5	6.68e-06
GO:0003937 IMP cyclohydrolase activity	**1**	1	9.44e-04
GO:0004044 amidophosphoribosyltransferase activity	**1**	1	9.44e-04
GO:0004637 phosphoribosylamine-glycine ligase activity	**1**	1	9.44e-04
GO:0004643 phosphoribosylaminoimidazolecarboxamide	**1**	1	9.44e-04

formyltransferase activity			
GO:0006188 IMP biosynthetic process	**1**	1	9.44e-04
GO:0004642 phosphoribosylformylglycinamidine synthase activity	**1**	2	1.89e-03
GO:0006189 'de novo' IMP biosynthetic process	**1**	7	6.60e-03
GO:0009116 nucleoside metabolic process	**1**	9	8.47e-03
GO:0006541 glutamine metabolic process	**1**	16	1.50e-02
GO:0046872 metal ion binding	**2**	344	3.53e-02
GO:0051539 4 iron, 4 sulfur cluster binding	**1**	48	4.46e-02

File S1. Tiling arrays data for primary (*B. subtilis* 168) network reconstruction. URL: https://figshare.com/s/04da90f4b22822476d98, DOI: 10.6084/m9.figshare.5277787.

File S2. RNA-Seq data (averaged replicates per condition) for secondary *B. subtilis* 168 network reconstruction. URL: https://figshare.com/s/50a269eb08fd4c634608, DOI: 10.6084/m9.figshare.5285461.

File S3. RNA-Seq data (averaged replicates per condition) for secondary *B. amyloliquefaciens* network reconstruction. URL: https://figshare.com/s/f8a622c8fcc924f50b08, DOI: 10.6084/m9.figshare.5285278.

File S4. Hub genes from the *B. subtilis* 168 co-expression network. URL: https://figshare.com/s/e41357c49624f461955e, DOI: 10.6084/m9.figshare.5099911.

File S5. Hub genes from the *B. amyloliquefaciens* co-expression network. URL: https://figshare.com/s/2befdedb49a4ef1bb857, DOI: 10.6084/m9.figshare.5099905.

References

(1) Buescher JM, Liebermeister W, Jules M, Uhr M, Muntel J, Botella E, et al. Global network reorganization during dynamic adaptations of Bacillus subtilis metabolism. Science 2012 Mar 2;335(6072):1099-1103.
